# The Condition of Midwives

**Published:** 1887-08-06

**Authors:** E. A. Bedingfeld

**Affiliations:** Lady Superintendent, Belgravia Midwives' Institute


### The Condition of Midwives.

By Mrs. E. A. Bedingfeld, Lady Superintendent, Belgravia Midwives' Institute.

Seeing that The Hospitals Association purposes during its next session taking into consideration the Amelioration of the Condition of Midwives, may I, as a midwife in practice, say a few words on this important subject, and state a few facts that may be new to some of your readers?

I think few are aware of the number of women who are annually, in the United Kingdom, attended by midwives of some sort. It was estimated some years ago that their number exceeded one million per annum. It was also calculated that for these to be efficiently looked after, one midwife was needed to every 2,000 of the population. This means, that reckoning the population of England and Wales (not the United Kingdom) at 23,000,000 (Whitaker gives it as 25,974,439 in 1881), there would be 11,500 midwives required in these two countries alone! I need hardly say that there are not nearly this number of trained midwives. Therefore it follows that a large number of women must be dependent on the services of women without skill or training. The consequences of this unskilled attendance are in many cases most serious, if not fatal, and serve to fill the women's wards of our hospitals. Such a state of affairs is a disgrace to any Christian country.

The United Kingdom of Great Britain and Ireland, I believe of all the countries in Europe, alone enjoys the enviable (?) distinction of not providing State legislation regarding midwives. In foreign lands the midwives are trained by the State, and work under Government control, no untrained woman being allowed to practise as a midwife. But in civilised England any woman, no matter how ignorant or drunken, may call herself a midwife, and practise as such, endangering the lives of others without let or hindrance. It is only when some startling case of death occurs, and an inquest is held, that the public are roused for a while to the evil that is going on, to get as apathetic in the matter as ever when the case blows over. That the evil has been long recognised is shown by the fact that two hundred and fifty years ago Peter Chamberlen wrote praying "that some order may be settled by the State for the instruction and civil government of midwives,"—and still the same cry goes up. In 1813 the Society of Apothecaries made an attempt "to persuade Parliament to pass enactments for the examining and control of midwives"; but in the Transactions of the Society of Apothecaries it is recorded, *apropos* of their attempt, that "the Committee of the House of Commons would not allow any mention of female midwives." In 1869 the Council of the Obstetrical Society of London appointed a committee to inquire into the causes of infant mortality, and to ascertain the proportion of cases attended by medical men and midwives, and to the instruction, if any, that the midwives received. The result was the establishment by the Society (in 1872, I think) of the quarterly examination of midwives, which is still held by them.*

But more is wanted, and some six or seven years ago a few ladies, stirred by the knowledge of what their poorer sisters were suffering at the hands of these "handy women," as they are in some places called, met together to see what could be done. The result was that the "Matrons' Aid Society and Midwives' Institute" was started, its object being to promote the better education of midwives, and to urge on a Bill providing for their registration and licensing by Act of Parliament. The Society still exists, and is doing, I believe, good work, but alas! the special object of its desires is yet unaccomplished. In 1882 a draft Bill was drawn up by the Obstetrical Society of London, and was submitted by them to the then Lord President of the Privy Council (Lord Carlingford), and receiving his approval, was by him passed on to the General Medical Council, who also approved of its aims. The Bill, however, got no further, though a large number of petitions in its favour were presented in Parliament at that time. The Bill, as then drawn, provides, among other things, that any midwife who can bring satisfactory proof of having been in *bonâ-fide* practice for the year previous to the passing of the Bill, and can produce the certificates of character, etc., required by the Bill, shall be entitled to be registered. Those who cannot fulfil the first of these conditions will have to pass an examination instituted by a Midwifery Board appointed by Government. The Bill also renders it penal for any woman to practise as or call herself a midwife without a licence.

If this Bill were passed the tone of midwives would be raised at once, as it would put out of the field all the ignorant and unqualified women who now take the work from those more educated ones who have given their time and money, in order to thoroughly learn as much as is taught of their profession, and the stigma that has so long been attached to the calling of a midwife being removed, a better class will go in for being trained. Already matters are improving, and the number of those midwives who go up for the Obstetrical Society's examination has largely increased during the last few years. But not until the Bill has passed, and the poorer classes understand that no woman is allowed to practise as a midwife without a licence, any more than a cabman is allowed to drive without one, will the calling take the place among occupations for women that it should do. It has fallen into disrepute, through the characters and ignorance of so many who have plied it, and, until lately, educated women have been deterred from training, because it was thought to be a "low trade." In this the wealthy women of our land might help by example. If some of them would put their prejudice aside, and now and again employ a properly qualified midwife for themselves, the prejudice would speedily be overcome: and many a hard-worked medical man would be thankful to have that most laborious, because most tedious, portion of his practice taken off his hands. (I have often wondered, *apropos* of this, that doctors do not use midwives more than they do to watch lingering cases for them, and so save themselves many wasted hours.) There are some

* For some of these facts I am indebted to an interesting little work by Dr. J. H. Aveling, entitled *English Midwives, their History and Prospects.*

ladies courageous enough to trust themselves to midwives, and I hope their numbers will increase.

When the Bill becomes law the question of more and cheaper schools of midwifery will arise, and also that of the higher education of midwives. The present training is too expensive for many who would otherwise adopt the calling. But it seems to me that if the Boards of Guardians would open the lying-in wards of the large infirmaries as midwifery schools, the necessary tuition fees might be greatly reduced. For the present the thing is to press on legislation, by all the means in our power, and leave other things to be entered into and grappled with when the first great good is obtained. If The Hospitals Association will use its influence in aid of this, it will be doing a good work, not only towards those who employ midwives, but also for the midwives themselves.

